# Comparing health gains, costs and cost-effectiveness of 100s of interventions in Australia and New Zealand: an online interactive league table

**DOI:** 10.1186/s12963-022-00294-3

**Published:** 2022-07-27

**Authors:** Natalie Carvalho, Tanara Vieira Sousa, Anja Mizdrak, Amanda Jones, Nick Wilson, Tony Blakely

**Affiliations:** 1 Health Economics Unit, Centre for Health Policy, School of Population and Global Health, The University of Melbourne, Level 4, 207 Bouverie St, Parkville, VIC 3010 Australia; 2 Music Therapy, Faculty of Fine Arts and Music, The University of Melbourne, Melbourne, Australia; 3 Burden of Disease Epidemiology, Equity, and Cost-Effectiveness Programme (BODE3), Department of Public Health, University of Otago, Wellington, New Zealand; 4 Population Interventions Unit, Centre for Epidemiology and Biostatistics, School of Population and Global Health, The University of Melbourne, Melbourne, Australia

**Keywords:** Cost-effectiveness, League table, Priority-setting, Costs, Health gains

## Abstract

**Background:**

This study compares the health gains, costs, and cost-effectiveness of hundreds of Australian and New Zealand (NZ) health interventions conducted with comparable methods in an online interactive league table designed to inform policy.

**Methods:**

A literature review was conducted to identify peer-reviewed evaluations (2010 to 2018) arising from the Australia Cost-Effectiveness research and NZ Burden of Disease Epidemiology, Equity and Cost-Effectiveness Programmes, or using similar methodology, with: health gains quantified as health-adjusted life years (HALYs); net health system costs and/or incremental cost-effectiveness ratio; time horizon of at least 10 years; and 3% to 5% discount rates.

**Results:**

We identified 384 evaluations that met the inclusion criteria, covering 14 intervention domains: alcohol; cancer; cannabis; communicable disease; cardiovascular disease; diabetes; diet; injury; mental illness; other non-communicable diseases; overweight and obesity; physical inactivity; salt; and tobacco. There were large variations in health gain across evaluations: 33.9% gained less than 0.1 HALYs per 1000 people in the total population over the remainder of their lifespan, through to 13.0% gaining > 10 HALYs per 1000 people. Over a third (38.8%) of evaluations were cost-saving.

**Conclusions:**

League tables of comparably conducted evaluations illustrate the large health gain (and cost) variations per capita between interventions, in addition to cost-effectiveness. Further work can test the utility of this league table with policy-makers and researchers.

**Supplementary Information:**

The online version contains supplementary material available at 10.1186/s12963-022-00294-3.

## Background

All jurisdictions have constraints on what preventive and other health services are provided, such as policy and public support, fixed health budgets or health sector capacity. Within these constraints, jurisdictions should aim to achieve maximal health benefits and, if possible, savings to future health expenditure—or at least a cost-effective return on investment, while accounting for other important criteria, including equity. Integrated epidemiological and economic evaluation studies provide estimates of future health gains, cost impacts, and cost-effectiveness of single or multiple interventions. If interventions fall below a pre-specified “willingness-to-pay (WTP) threshold” or are cost saving, they are considered good value for money and worthy of being introduced. However, the reliance on WTP thresholds alone to guide decision rules has been criticised, with the key problems identified being a lack of theoretical justification, the appropriate estimation of these thresholds, and the lack of accounting for other relevant decision-making considerations [1, 2]. For example, a fixed WTP threshold does not take budget considerations into account, and often interventions found to be “cost-effective” are not implemented due to budget impact constraints in that funding cycle [3]. On the health outcomes side, public health experts and policy-makers are often unaware of the magnitude of potential health gains from interventions; comparable estimates of future health gains and costs impacts across multiple preventive and other health interventions are needed to inform the prioritisation alongside cost-effectiveness.

One approach to prioritisation is league tables, pioneered by Alan William’s comparison of cardiovascular disease treatments in 1985 to identify which treatment was near ‘the top of the league’ [4]. Such league tables typically rank health interventions by cost per life year or cost per quality-adjusted life year gained. League tables were quickly criticised about the lack of methodological consistency in conduct of cost-effectiveness analysis (CEA), which could bias the ranking of interventions [5, 6]. Additionally, league tables may over-emphasise cost-effectiveness relative to the size of the health gain or cost-savings, addressing health inequalities, and intervention feasibility [5].

We see an opportunity for a new era of league tables. First, there has been progressed articulating best practice guidelines for the conduct and reporting of CEA (e.g. Consolidated Health Economic Evaluation Reporting Standards (CHEERS) checklist among others [7–9]), but also more generally in modelling the health impacts of preventive interventions [10]. Second, with the adoption of these guidelines, there are now higher-quality estimates from which to construct a more methodologically robust league table [11]. Third, online tools for presenting and interacting with data open the potential for user input to league table generation, and greater flexibility in the presentation of output.

However, intervention simulation and cost-effectiveness studies often lack comparability. For example, variations in discount rates, time horizons over which benefits are assessed, and perspectives can lead to difficulties in making fair comparisons of health benefit, cost, and value for money. There are limited examples of league tables comparing interventions from methodologically consistent evaluations; for example: obesity interventions [12, 13], tobacco endgame strategies [14], dietary sodium reduction interventions[15], a varied package of preventive interventions [16], and preventive and therapeutic interventions targeting non-communicable diseases [17]. Online repositories such as the Tufts New England CEA registry and Global Health CEA registry (www.cearegistry.org) contain thousands of cost-utility analyses conducted globally; however, there are no restrictions on the comparability of methods used in the studies and therefore limited confidence in comparability.

Australia and New Zealand (NZ) are fortunate to now have a large body of evaluations (primarily preventive but some treatment) conducted using a comparable proportional multi-state lifetable (PMSLT) method [18], spawned by the Assessing Cost-Effectiveness (ACE) studies in Australia—particularly the ACE-Prevention study [16, 19]—and now also by the NZ Burden of Disease Epidemiology, Equity and Cost-Effectiveness Programme (BODE^3^; www.otago.ac.nz/bode3). Briefly, the ACE-BODE^3^ methodology models interventions as applied to a specified population (those alive in the base-year), using a PMSLT populated with epidemiological inputs derived from a burden of disease study, using an unrelated cost perspective used (i.e. not just the costs of the disease or risk factor targeted, but including the costs of other diseases that the population may incur due to living longer), and health and cost impacts tallied up for the remainder of the population’s lifespan (unless stated otherwise). Results are presented as health gains, net health expenditure (i.e. the net of upfront intervention costs, and downstream cost-offsets) and incremental cost-effectiveness ratios using a cost-utility analysis approach [16]. This paper describes the collation of comparable evaluations from the ACE and BODE^3^ Programmes, published or in press from 2010 to 2018. We provide outputs in comparable units of health outcomes, costs, and cost-effectiveness. Furthermore, we give graphical examples of league table comparisons using an online interactive tool, the Australia and NZ Health Intervention League Table (ANZ-HILT).

## Methods

We compiled input data and health and economic outputs from evaluations from Australia and NZ conducted strictly following the ACE- BODE^3^ methodology, sourced from the following peer-reviewed publications:

- The Australian ACE Prevention Report [16], with evaluations replaced by peer-reviewed paper if available.
- Australian peer-reviewed papers and reports using ACE methodology [16, 18] published from 1 January 2010 to 31 December 2018, found by searching on key authors (details in Additional file 1: Appendix).
- NZ publications from the BODE^3^ Programme (www.otago.ac.nz/bode3).

The unit of presentation and analysis was an evaluation; a publication or report may include many evaluations of variants of the same intervention.

### Eligibility criteria

Evaluations were included if they met these criteria:

- A clear description of the intervention duration and, where appropriate, frequency.
- Quantified health impacts in health-adjusted life years (HALYs): either quality-adjusted life years (QALYs) gained or disability-adjusted life years (DALYs) averted.
- Quantified either:
  - at least two of the three following health system costs:
    - Intervention cost
    - Averted or incurred future health system cost offsets due to changing future disease incidence, including those costs unrelated to the diseases or conditions directly affected by the intervention.[8, 19]
    - Net costs (i.e. intervention costs minus cost offsets).
  - an incremental cost-effectiveness ratio (ICER) — with the numerator satisfying the ‘net cost’ definition above.
- At least a 10-year time horizon post-intervention commencement for the accrual of HALYs and costs.
- Applied a discount rate of between 3 and 5% for both health gains and costs.

(There are Australasian studies that meet the above eligible criteria that are not part of the ACE and BODE^3^ bodies of work; they are included at ANZ-HILT, but for parsimony not presented in this paper.)

### Data extracted from each evaluation

We extracted the following data from each evaluation: (1) setting, including country, description and size of target population, year in which intervention started, currency and base-year for costs; (2) intervention characterisation, including duration of intervention and frequency of intervention, along with comparator; (3) methods, including perspective of the analysis, time horizon of simulation follow-up and discount rate; (4) outcomes, including HALYs gained, costs, ICER, and 95% uncertainty intervals.

In cases where data were not explicitly provided in the main paper or Additional file 1, we estimated the total eligible target population (using official statistical agency data on population counts by, for example, age) and calculated the ICER from net costs and HALYs.

### Processing of extracted outputs and visualisation of evaluations

Evaluations were categorised into one of 14 domains derived from the Global Burden of Disease Study’s risk factor domains: alcohol; cancer; cannabis; communicable disease; cardiovascular disease (CVD); diabetes; diet; injury; mental illness; other non-communicable diseases (NCD); overweight and obesity; physical inactivity; salt (dietary); and tobacco.

Based on what population level the intervention was directed towards, each intervention was assigned to one of three categories: population-wide, intermediary ‘partial targeting’, or ‘targeted’. Population-wide represents interventions such as the reformulation of food and tobacco taxes. Partial targeting was any programme directed towards less than a quarter of the total population, such as school-based programmes, and captured most screening programmes and vaccination programmes. Targeted was any treatment intervention for people with a disease. This included rehabilitation and screening post-diagnosis but excluded secondary prevention among people with risk factors rather than a disease (e.g. hypertension or obesity; coded as ‘partial targeted’).

### An online, user-friendly visualisation tool

To assist interpretation and make evaluations flexibly available to interested users, we created a user-friendly R Shiny App tool (ANZ-HILT) to allow visualisation of evaluations (https://league-table.shinyapps.io/bode3/). ANZ-HILT allows interventions to be compared by HALYs gained, net costs and ICER (where the intervention is not cost-saving). Interventions were categorised as ‘cost-saving’ if net costs were negative and HALYs positive, and as ‘dominated’ if both the net costs were positive and HALYs negative, compared to the comparator scenario. HALYs and costs are shown as a total applied to the whole country and per 1000 people in the total population in the base-year. Costs are presented inflation and purchasing power parity (Organisation for Economic Co-operation and Development (OECD)) adjusted to any year between 2010 and 2016 in three currencies, United States dollars (US$), NZ dollars (NZ$) or Australian dollars (AU$). In the current paper, we give graphical examples of league table comparisons using this tool to showcase some of the possible comparisons across interventions. ANZ-HILT also contains additional evaluations and outputs beyond the scope of this paper.

## Results

A total of 384 evaluations met the inclusion criteria (see Additional file 1: Appendix). Of the 318 Australian evaluations, 94 were reported in the original ACE-Prevention Report and also published in a journal article (used as the primary source), 137 were published only in the Report. Beyond the original ACE-Prevention Report, 15 more evaluations arose from an obesity report and another 72 arose from 23 peer-reviewed articles. All 66 NZ evaluations came from 25 peer-reviewed articles arising from the BODE^3^ Programme.

We excluded three interventions deemed no longer relevant: circumcision of men to prevent HIV infection (not relevant in NZ and Australia) [16], and two cardiovascular disease polypill evaluations with a price of AU$5000 per year [16]—which is far higher than current pricing.

### Characteristics of included evaluations

Table 1 shows the characteristics of evaluations, by country. The majority of Australian evaluations were published before 2015 (77.7%) and used 2003 as the base-year, while the majority of NZ evaluations were published after 2015 (92.4%) and all used 2011 as the base-year. A health sector or health system perspective was used across the majority of evaluations (53.9%) and nearly all of NZ evaluations (97.0%). A societal or limited societal perspective was used in a minority of evaluations (7.8%), and multiple perspectives (including health sector, government, patient, and/or societal) were used in a small number of evaluations (6.8%). The perspective was not specified in nearly a third (30.5%) of evaluations with the majority of these being Australian evaluations from the ACE-prevention report, in which a health sector perspective was adopted for all evaluations unless non-health sector impacts were found to be important. All NZ evaluations used a lifetime perspective, and only 4.1% of the Australian evaluations had less than a lifetime perspective—and these were mostly mental health and communicable diseases interventions. All evaluations used a 3% discount rate. The majority of Australian evaluations were targeted (8.2%) or partially targeted (56.6%), while the NZ evaluations were mostly population-wide (72.7%). The large majority of evaluations in both countries (92.4%) were preventive. Over half (57.3%) of evaluations across both countries were for interventions that persisted over the remainder of the population’s life span (e.g. tax interventions) with the second most common intervention duration being one-off or up to 1 year (20.8% overall). Over half of Australian evaluations were related to cardiovascular disease (29.6%), overweight & obesity (14.2%) and diet (13.5%). The NZ evaluations were more concentrated by domain, with 81.8% being from one of three domains (cancer, dietary salt and tobacco). The most common comparator was current practice (or business-as-usual) (43.5%), followed by “Do nothing” (37.2%), which most was usually the same thing as current practice. Only 1.3% of evaluations had a different comparator specified, and 18.0% of evaluations did not specify a comparator, with the majority of those (68/69) being Australian evaluations.

**Table 1:** Characteristics of included evaluations

	Australia		New Zealand		Total	
	N	%		%		%
Total evaluations	318		66		384	
Year published						
2010–2014	247	77.7%	5	7.6%	252	65.6%
2015–2018	71	22.3%	61	92.4%	132	34.4%
Base-year in model						
2000–04	207	65.1%	0	0%	207	53.9%
2005–09	40	12.6%	0	0%	40	10.4%
2010–15	71	22.3%	66	100%	137	35.7%
Perspective						
Health sector/Health system	143	45.0%	64	97.0%	207	53.9%
Government	4	1.3%	0	0%	4	1.0%
Multiple (Health sector, Government, Patient, Societal)	26	8.2%	0	0%	26	6.8%
Societal (or limited societal)	30	9.4%	0	0%	30	7.8%
Not specified^	115	36.4%	2	3.0%	117	30.5%
Time horizon						
10y to < lifetime	13	4.1%	0	0%	13	3.4%
Lifetime	95	95.9%	66	100%	371	96.6%
Discount rate (annual)						
3%	318	100%	66	100%	384	100%
Other	0	0%	0	0%	0	0%
Degree of targeting						
Population-wide	112	35.2%	48	72.7%	160	41.7%
Partially targeted	180	56.6%	14	21.2%	194	50.5%
Targeted	26	8.2%	4	6.1%	30	7.8%
Intervention duration						
One-off or up to 1 year	64	20.1%	16	24.2%	80	20.8%
1–5 years	9	2.8%	1	1.5%	10	2.6%
6–20 years	20	6.3%	1	1.5%	21	5.5%
Persistent	172	54.3%	48	72.7%	220	57.3%
Not specified	53	16.7%	0	0%	53	13.8%
Type of intervention						
Prevention	298	93.7%	57	86.4%	355	92.4%
Treatment	20	6.3%	4	6.1%	24	6.3%
Missing	0	0%	5	7.6%	5	1.3%
Type of comparator^+^						
Current practice (Business-as-usual)	137	43.1%	30	45.5%	167	43.5%
Do nothing	111	34.9%	32	48.5%	143	37.2%
Other	2	0.6%	3	4.5%	5	1.3%
Not specified	68	21.4%	1	1.5%	69	18.0%
Domain						
Cancer	27	8.5%	8	12.1%	35	9.1%
Alcohol	16	5.0%	0	0%	16	4.2%
Cannabis or other illicit drugs	5	1.6%	0	0%	5	1.6%
Communicable disease	7	2.2%	5	7.6%	12	3.1%
Cardiovascular disease	94	29.6%	1	1.5%	95	24.7%
Diabetes	13	4.1%	0	0%	13	3.4%
Diet	43	13.5%	0	0%	43	11.2%
Injury	1	0.3%	5	7.6%	6	1.6%
Mental illness	8	2.5%	0	0%	8	2.1%
Other NCD	26	8.2%	0	0%	26	6.8%
Overweight & obesity	45	14.2%	1	1.5%	46	12.0%
Physical activity	16	5.0%	0	0%	16	4.2%
Salt (dietary)	3	0.9%	32	48.5%	35	9.1%
Tobacco	14	4.4%	14	21.2%	28	7.3%
*Health gain*						
HALYs per 1000 total population						
< 0.10	122	28.4%	8	12.1%	130	33.9%
0.10–1	86	27.0%	2	3.0%	88	22.9%
1–10	77	24.2%	30	45.5%	107	27.9%
> 10	28	8.8%	22	33.3%	50	13.0%
Missing^†^	5	1.6%	4	6.1%	9	2.3%
HALYs per person in target population						
< 01	28	8.8%	23	34.9%	51	13.3%
01–099	0	0%	20	30.3%	20	5.2%
0.1–0.99	1	0.3%	3	4.5%	4	1.0%
Missing^†^	289	90.9%	20	30.3%	309	80.5%
*Incremental health expenditure*						
Net cost* per 1000 total population						
< US$0 [Cost saving]	103	32.4%	46	69.7%	149	38.8%
US$0 to $10,000	99	31.2%	9	13.6%	108	28.1%
Cost > US$10,000	67	21.1%	8	12.1%	75	19.5%
Missing^†^	49	15.4%	3	4.6%	52	13.5%
Net cost* per target population						
< US$0 [Cost saving]	1	0.3%	32	48.5%	33	8.6%
US$0 to $1000	24	7.6%	10	15.2%	34	8.9%
Cost > US$1000	4	1.3%	2	3.0%	6	1.6%
Missing^†^	289	90.9%	22	33.3%	311	81.0%
*COST per HALY or Incremental cost-effectiveness ratio*						
Cost saving	97	30.5%	47	71.2%	144	37.5%
US$0 to $50,000 per HALY	127	39.9%	17	25.8%	144	37.5%
> US$50,000 per HALY	82	25.8%	1	1.5%	83	21.6%
Dominated	3	0.9%	0	0%	3	0.8%
Missing^†^	9	2.8%	1	1.5%	10	2.6%

^*^2016 US$

^ Most evaluations in which the perspective is listed as “Not specified” are from the original ACE-prevention report. According to the methodology stated, a “health sector perspective” is adopted for all evaluations unless non-health sector impacts were deemed important and then captured through a sensitivity analysis

+ Most evaluations reporting “Do nothing” as comparator used the current status in the absence of the intervention as comparator, rather than stripping back current interventions in place. Many studies in which the comparator was not stated in fact also appeared to have the current status (or no intervention) as comparator

^†^ Most studies reported results for HALYs and costs for either a total population perspective (e.g. for all eligible people in Australia) or a per capita perspective — but not both (although we were able to sometimes calculate both if sufficient data were reported in the paper)

The majority of evaluations across both countries (97.7%) were able to have results expressed for the total population, whereas only a minority (19.5%) were able to have results expressed per capita of a target population. The distribution of HALYs gained and net costs per 1000 of the total population and per capita of the target population is shown in Table 1. Most (71.2%) of the NZ evaluations were cost-saving, but only 30.5% of the Australian ones were. Conversely, 26.7% of the Australian evaluations had either an ICER > US$50,000 (beyond the rule of thumb of Gross Domestic Product (GDP) per capita being a threshold beyond which interventions are deemed not cost-effective) or were dominated (i.e. performed worse than the comparator), compared to only 1.5% of the NZ evaluations.

### Selected examples of ANZ-HILT outputs

Figure demonstrates a histogram output from ANZ-HILT showcasing a selection of interventions. The left-hand panel of ANZ-HILT allows the user to select permutations of: domain (three here: cancer, diet and tobacco), actual interventions (seven here), currency and year to show dollars in, range of publication years that the evaluation was published in, and the outcome variable to plot (HALYs here). The tabs: across the top allow the user to view instructions, population denominator (here per 1000 of the total population), or cost-effectiveness plane (see Fig. 2 below); and beneath toggle between table and ‘plot top 10’ options (plot shown here). A hover-over with one’s computer mouse allows the user to see the following for each evaluation: expected number of HALYs/discount rate/time horizon of follow-up/comparator/and intervention duration and/or frequency. For example, for the 10% per annum tobacco tax intervention, hovering over the bar will cause the following text to appear: “53,200/0.03/Lifetime/Business-as-usual (no tax increases from 2011-to 2025)/14 years of tax increases, then persistent”. Further information can be found in the table tab, e.g. the actual lower and upper uncertainty limits.

Turning to the substantive patterns in Figure, there is an over 1000-fold variation from 23 per 1000 (95% uncertainty interval (UI): 18 to 29) HALYs gained over the remainder of life for NZ population aged 35 + years in 2011 for 25% of salt in processed food being replaced by potassium and magnesium salts (where that intervention is ‘left on’ for the remainder of the population’s lifespan), down to 0.019 per 1000 (95% UI: 0.011 to 0.029) for all stage III colon cancer patients diagnosed in 2011 being assisted by cancer care coordinators to navigate more quickly and with higher coverage to surgery and chemotherapy. With this (and other intervention combinations), differences in the timespan and target groups of interventions often account for differences in the magnitude of health gains.

This variability in intervention conceptualisation noted, we can make some inferences from the selected examples shown Figure:

- 25% salt substitution with potassium and magnesium salts (NZ) and food taxes across saturated fat, excess salt, sugar-sweetened beverages and sugar in processed foods (Australia) leads to similarly large health gains.
- Interventions such as ‘Tick’ logos on health food and dietary advice for those with high blood pressure have much smaller health gains when summed up across the population.

**Fig. 1:**
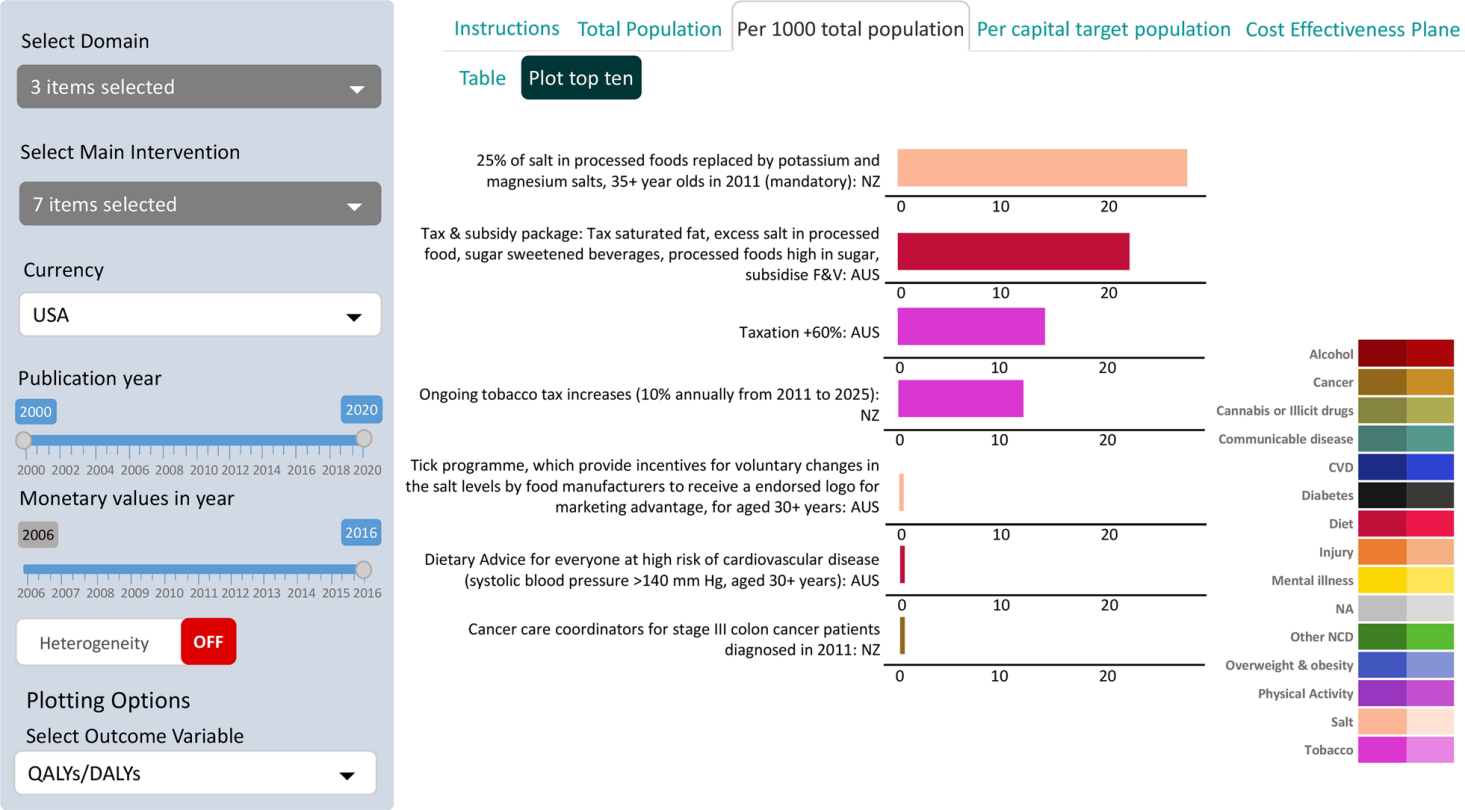
HALYs gained per 1000 in the total population, for selected Australian and New Zealand health sector interventions

The health system expenditure impacts of the interventions shown in Fig. 1 are correlated—but with large cost-savings (due to future disease rates being considerably lower) for interventions with large health gains (Additional file 1: Figure S1). Intervention impacts can also be presented in terms of HALYs gained per capita in the target population (Additional file 1: Figure S2).

**Fig. 2:**
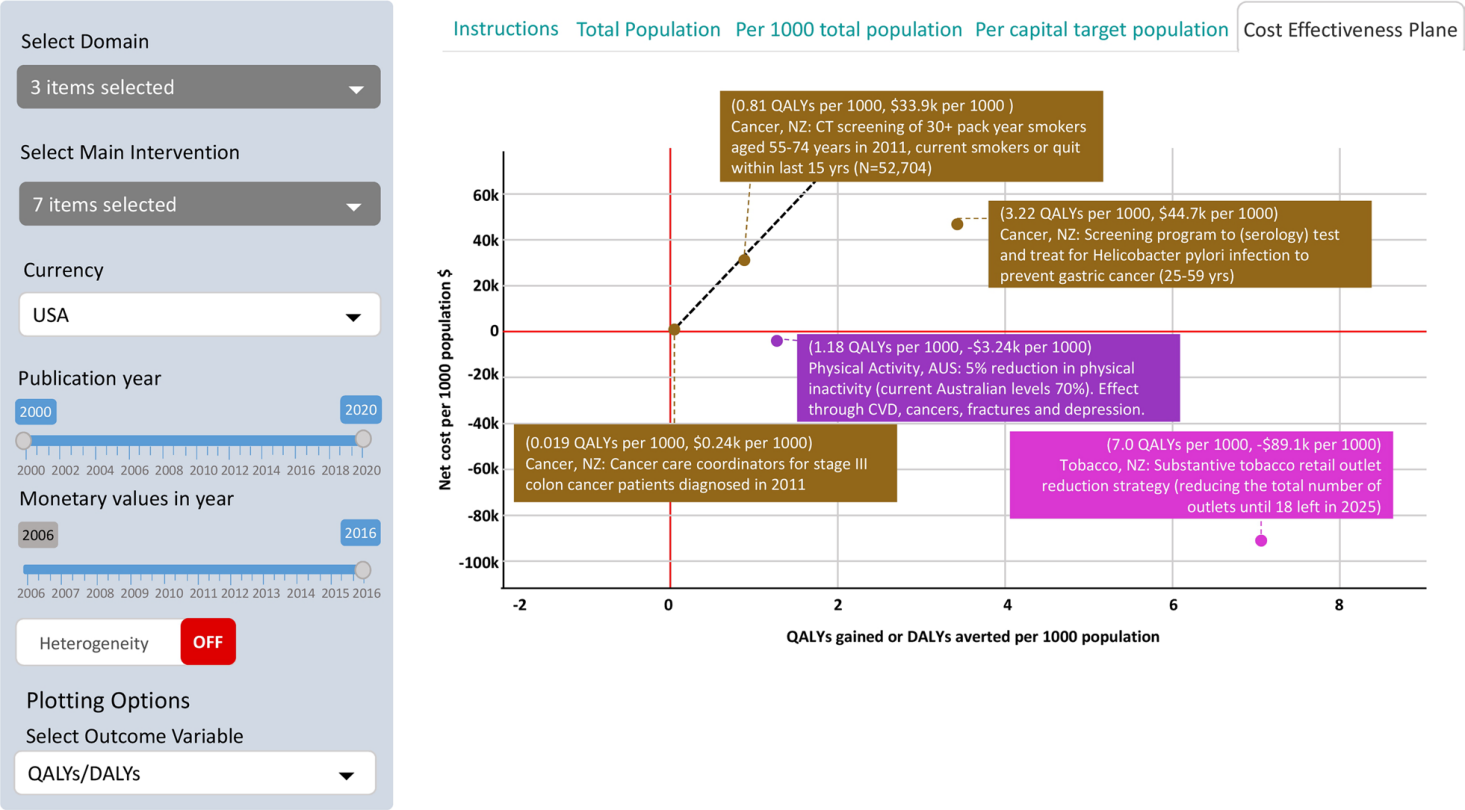
Cost-effectiveness plane for selected interventions. Confidence intervals (CI) about the QALYs gained and net costs are in tabular output at ANZ-HILT

Figure 2 is an example of a cost-effectiveness plane output for five evaluations, with two overlays: the text pop-ups that appear as the user hovers over each point are shown for all five interventions; the black dashed line is a super-imposed threshold line at about GDP per capita per HALY. The plane now allows the user to simultaneously see (often massive) variation between interventions in all of health gain, cost and cost per HALY gained. For this example, we present results in 2016 US$. For example, the tobacco retail outlet reduction intervention is in the southeast quadrant with substantial health gains (7 HALYs per 1000 over the remaining lifespan of the population) and cost-savings (US$89,100 per 1000). On effectiveness and efficiency grounds alone, this intervention should be considered for implementation — but there are other considerations such a political will and societal preferences that are not captured in ANZ-HILT. The *Helicobacter pylori* screening programme (to detect infection that is then treated with antibiotics, reducing stomach cancer incidence rates years into the future) is in the northeast quadrant, but beneath the willingness to pay line (black dashed line) suggesting it is cost-effective at a GDP per capita threshold. The computed tomography (CT) screening of heavy smokers is also in the northeast quadrant costing US$42,000 per QALY gained (i.e. 33.9/0.81) — about the threshold GDP per capita per QALY gained.

## Discussion

This paper demonstrates the consolidation of key health and economic outputs from hundreds of comparably conducted evaluations for Australia and NZ in a world first online interactive league table. ANZ-HILT allows for the comparison of health gain, net cost and cost-effectiveness of (mostly) preventive interventions across a wide range of domains. While only 6% of evaluations included in this paper are for treatment of disease, with the majority focused on prevention, this is an artefact of the focus areas of the research group(s) conducting the evaluations, and the ACE methodology can still be applied more widely for treatment-related interventions. The league table discloses large differences in impact between interventions. Such information has hitherto not been easily accessible by stakeholders and end-users. Our aim is to better inform policy-makers and health experts, who are often unaware of the relative magnitude of intervention impact.

### Early indications of benefits and barriers for end-users

We have some evidence of utility and impact to date. First, a league table of dietary interventions was the backbone to discussions requested by the NZ Minister of Health on food reformulation options, as he was in parallel in negotiation with the food industry. Second, key informant interviews of 16 senior policy-makers in Australia by an independent consult (commissioned by us as part of other work, February to March 2020, unpublished) found strong support for greater information of the type shown in ANZ-HILT: “There was general agreement that the lack of robust, comparable, and easy-to-access data on the impacts of various health programmes hinders the design and prioritisation of public health interventions”. However, there were also barriers to uptake identified, including a culture in policy making that was not always receptive to such evidence (e.g. due to time, capacity or other reasons), that will require addressing beyond the simple publication of a tool such as ANZ-HILT (e.g. outreach and championing).

### Strengths and limitations

The strengths of ANZ-HILT include the selection of evaluations meeting comparability criteria, and visualisations through graphs that focus not only on incremental cost-effectiveness—but also net heath gains and costs. There are also many limitations. First, we can only include what has been evaluated. It would be a false conclusion that because dietary interventions in ANZ-HILT tend to have lower *health* gains per 1000 population that we expect all dietary interventions to be similar; it depends on what specific evaluation researchers chose. There may be other more effective and less costly interventions that were excluded from ANZ-HILT because they used a different methodology. However, we cannot reliably tell if another evaluation is more effective or less costly, unless it uses the same methodology—hence, the primacy we afford to only including comparable studies. Second, among all the studies actually conducted, there may be non-ACE-BODE^3^ studies that met our eligibility criteria; the few such Australasian studies are included at the ANZ-HILT tool, but not in this paper for parsimony. Non-Australasian studies were out of scope, but if there was interest, collaboration and funding from other countries ANZ-HILT could be extended to Global-HILT. Third, we use explicit criteria to select comparable evaluations, but we do not further restrict based on the quality of evaluation. Rather, we necessarily use a *caveat emptor* or ‘user beware’ approach—facilitated by provision of URL links to the underlying published evaluations. Fourth, the coding of some of the data extracted from evaluations is imprecise, most notably specifications of comparator, perspective, and whether the intervention was population-wide or partially targeted. While a substantial proportion (18%) of evaluations did not specify the comparator, in many cases, the comparator was the current status and is a reflection of incomplete reporting standards. While nearly one third (30.5%) of evaluations did not specify the perspective, in most cases, these evaluations were from the original ACE-prevention report, in which the perspective was that of the health sector. However, this was not always the case, as for example, breath testing for alcohol may have included broader societal costs. Regarding targeting, while we regard the presentation of HALYs and net costs per capita is a useful goal and part of the functionality in ANZ-HILT, determining the correct ‘target’ population is often challenging. For example, is the target population for a smoking cessation programme: all smokers, just those who are open to the idea of quitting, or only those planning a quit attempt? Lastly, there are considerations beyond health benefits, costs and cost-effectiveness that are not included in ANZ-HILT, yet that may be relevant to policy makers including equity impacts of interventions.

Despite these limitations, we believe that ANZ-HILT provides a first step towards providing a publicly available, easy-to-use online tool that consolidates some of the key aspects that are important to inform decision-making by policy-makers and health experts. We envision keeping this online tool up-to-date with new evaluations added in each year, similarly to the Tufts New England CEA and Global Health CEA registries, subject to collaborations and funding. As has been demonstrated by the WHO-CHOICE group [20], evaluations conducted similarly can still be compared across different healthcare settings and countries, and we believe that there is scope to do the same with a Global-HILT. This would provide something similar to the CEA registries, but with a narrower scope of similarly conducted evaluations to ensure comparability across evaluations. As countries move towards more standardised guidance for cost-utility analysis, we believe more evaluations will be available to consolidate within a Global-HILT that could be useful to policy-makers beyond Australia and New Zealand.

## Conclusions

League tables will never provide all the information necessary for policymakers to make prioritisation decisions, but they could be an excellent starting point for deliberation. Further research could probe validity (e.g. age-standardisation of outputs, comparability of costing methods) and quality (e.g. CHEERS checklist); extend outputs (e.g. health gains and net costs in first 10 and 10–20 years post-intervention); include compatible evaluations from other countries; and further research utility and impact with end-users including policy-makers and researchers. We propose that other researchers and countries consider contributing to a global-HILT.

## Supplementary Information

**Additional file 1.** Appendix.

## Data Availability

All data generated and analysed during this study are available from the corresponding author upon request.

## Abbreviations

ACE: Australia cost-effectiveness
ANZ-HILT: Australia and NZ health intervention league table
AU$: Australian dollars
BODE3: Burden of disease epidemiology, equity and cost-effectiveness
CHEERS: Consolidated health economic evaluation reporting standards
CEA: Cost-effectiveness analysis
CT: Computed tomography
CVD: Cardiovascular disease
GDP: Gross domestic product
HALYs: Health-adjusted life years
ICER: Incremental cost-effectiveness ratio
NCD: Non-communicable disease
NZ: New Zealand
NZ$: NZ dollars
OECD: Organisation for economic co-operation and development
PMSLT: Proportional multi-state lifetable
US$: United States dollars
WTP: Willingness-to-pay
