# Supplementary material for: Comparing health gains, costs and cost-effectiveness of 100s of interventions in Australia and New Zealand: an online interactive league table
Source: Popul Health Metr. 2022 Jul 27;20:17. doi: 10.1186/s12963-022-00294-3 (PMC9327210; doi:10.1186/s12963-022-00294-3)
Supplement: Supplementary file 1 — Additional file 1. Appendix. [file 12963_2022_294_MOESM1_ESM.docx]

Appendix for “**Comparing health gains, costs and cost-effectiveness of 100s of interventions in Australia and New Zealand: an online interactive league table**”

Contents

[Literature review strategy for Australian peer-reviewed publications 2](#_Toc72761541)

[Publications with at least one included evaluation in this study 3](#_Toc72761542)

[Supplementary figures 9](#_Toc72761543)

[Examples of (interactive) leagues tables that already exist 10](#_Toc72761544)

[References 12](#_Toc72761545)

# Literature review strategy for Australian peer-reviewed publications

The literature review started with the evaluations documented in the ACE-Prevention Report. If the evaluation was subsequently published as a journal article, the extracted data were obtained directly from the original journal article– as occasionally data was updated. Evaluations based on journal articles published prior to 2010 were excluded. Otherwise we used the outputs published in the original Report. The literature search on PUBMED was based on the study authorship, listed in the Report appendices. From 231 evaluations originally published in the ACE-Prevention Report and analysed for this study, 94 were published in subsequent peer-reviewed journals (Figure 1 in main paper).

Evaluations not included in the ACE-Prevention Report, but subsequently published in peer reviewed journal up to and including 2018, using ACE methodology (and often including authors of the original Report) were also included following the research by authorship and a third search.

We used a third search to capture Australian evaluations not using the ACE-Prevention methodology, based on the PUBMED the strategy for articles published from 2010:

*(QALY$ or HALY or DALY or quality adjusted life year* or disability adjusted life year*)
 AND
 ((cost and effectiveness and analysis) or cost effective* or (cost and utility and analysis) or cost utility or cost benefit$ or (economic and evaluation) or economic evaluation)
 AND
 Australia* or New Zealand.*

From the initial search, 704 abstracts were read, and 172 potential articles identified for full text review. Data were extracted from 23 articles using ACE-Prevention methodology and incorporated into the ANZ-HILT (72 evaluations). (Additional articles and evaluations not using the ACE methodology were eligible and included in ANZ-HILT, but not included in this study.)

A report published in December 2018 (Assessing Cost-Effectiveness of Obesity Prevention Policies in Australia) was also included adding 15 more evaluations (Figure 1).

A list of included publications is provided below.

# Publications with at least one included evaluation in this study

| *ACE-Prevention Report as primary source* |
| --- |
| Vos T, Carter R, Barendregt J, et al. Assessing Cost-Effectiveness in the Prevention (Ace-Prevention): Final Report: University of Queensland and Deakin University, 2010. |
| *Initially in ACE-Prevention Report, but subsequently published in a peer reviewed journal article that was used as primary source (between 2010 and 2014)* |
| Byrnes JM, Cobiac LJ, Doran CM, et al. Cost-effectiveness of volumetric alcohol taxation in Australia. *The Medical journal of Australia* 2010;192(8):439-43. [published Online First: 2010/04/21]  Cobiac LJ, Magnus A, Barendregt JJ, et al. Improving the cost-effectiveness of cardiovascular disease prevention in Australia: a modelling study. *BMC public health* 2012;12:398.  Cobiac LJ, Vos T. Cost-effectiveness of extending the coverage of water supply fluoridation for the prevention of dental caries in Australia. *Community Dentistry & Oral Epidemiology* 2012;40(4):369-76.  Cobiac LJ, Vos T, Veerman JL. Cost-effectiveness of interventions to promote fruit and vegetable consumption. *PLoS ONE [Electronic Resource]* 2010;5(11):e14148.  Cobiac LJ, Vos T, Veerman JL. Cost-effectiveness of interventions to reduce dietary salt intake. *Heart (British Cardiac Society)* 2010;96(23):1920-5.  Cobiac L, Vos T, Veerman L. Cost-effectiveness of Weight Watchers and the Lighten Up to a Healthy Lifestyle program. *Australian & New Zealand Journal of Public Health* 2010;34(3):240-7.  Forster M, Veerman JL, Barendregt JJ, et al. Cost-effectiveness of diet and exercise interventions to reduce overweight and obesity. *International Journal of Obesity* 2011;35(8):1071-8.  Higashi H, Barendregt JJ. Cost-effectiveness of total hip and knee replacements for the Australian population with osteoarthritis: discrete-event simulation model. *PloS one* 2011;6(9):e25403. doi: 10.1371/journal.pone.0025403 [published Online First: 2011/10/04]  Mihalopoulos C, Vos T, Pirkis J, et al. The population cost-effectiveness of interventions designed to prevent childhood depression. *Pediatrics* 2012;129(3):e723-30. doi: 10.1542/peds.2011-1823 [published Online First: 2012/02/09]  Mogasale V, Barendregt J. Cost-effectiveness of influenza vaccination of people aged 50-64 years in Australia: results are inconclusive. *Australian and New Zealand journal of public health* 2011;35(2):180-6. doi: 10.1111/j.1753-6405.2010.00639.x [published Online First: 2011/04/06]  Mogasale V, Vos T. Cost-effectiveness of asthma clinic approach in the management of chronic asthma in Australia. *Australian and New Zealand journal of public health* 2013;37(3):205-10. doi: 10.1111/1753-6405.12060 [published Online First: 2013/06/05]  Ong KS, Carter R, Vos T, et al. Cost-effectiveness of interventions to prevent cardiovascular disease in Australia's indigenous population. *Heart, lung & circulation* 2014;23(5):414-21. doi: 10.1016/j.hlc.2013.10.084 [published Online First: 2013/11/21]  Sacks G, Veerman JL, Moodie M, Swinburn B. ‘Traffic-light’ nutrition labelling and ‘junk-food’ tax: a modelled comparison of cost-effectiveness for obesity prevention. [Int J Obes (Lond).](https://www.ncbi.nlm.nih.gov/pubmed/21079620) 2011 Jul;35(7):1001-9. doi: 10.1038/ijo.2010.228. Epub 2010 Nov 16.  Veerman JL, Barendregt JJ, Forster M, Vos T. Cost-effectiveness of pharmacotherapy to reduce obesity. *PLoS One*.2011 ;6(10):e26051. doi:10.1371/journal.pone.0026051 |
| *ACE methodology evaluation, not in ACE-Prevention Report, published in a peer reviewed journal 2010-18 inclusive* |
| Ananthapavan J, Sacks G, Brown V, et al. Assessing Cost-Effectiveness of Obesity Prevention Policies in Australia 2018 (ACE-Obesity Policy). Melbourne: Deakin University, 2018.  Ananthapavan J, Moodie M, Haby M, et al. Assessing cost-effectiveness in obesity: laparoscopic adjustable gastric banding for severely obese adolescents. *Surgery for Obesity & Related Diseases* 2010;6(4):377-85.  Brown V, Moodie M, Cobiac L, et al. Obesity-related health impacts of active transport policies in Australia - a policy review and health impact modelling study. *Australian and New Zealand journal of public health* 2017;41(6):611-16. doi: 10.1111/1753-6405.12726 [published Online First: 2017/10/19]  Brown V, Moodie M, Cobiac L, et al. Obesity-related health impacts of fuel excise taxation- an evidence review and cost-effectiveness study. *BMC public health* 2017;17(1):359. doi: 10.1186/s12889-017-4271-2 [published Online First: 2017/05/05]  Cadilhac DA, Cumming TB, Sheppard L, et al. The economic benefits of reducing physical inactivity: an Australian example. *The international journal of behavioral nutrition and physical activity* 2011;8:99. doi: 10.1186/1479-5868-8-99 [published Online First: 2011/09/29]  Cadilhac DA, Sheppard L, Cumming TB, et al. The health and economic benefits of reducing intimate partner violence: an Australian example. *BMC public health* 2015;15:625. doi: 10.1186/s12889-015-1931-y [published Online First: 2015/07/15]  Chew DP, Carter R, Rankin B, et al. Cost effectiveness of a general practice chronic disease management plan for coronary heart disease in Australia. *Australian Health Review* 2010;34(2):162-9.  Cobiac L, Tam K, Veerman L, Blakely T. Taxes and subsidies for improving diet and population health in Australia: A cost-effectiveness modelling study. PLoS Med 2017;14(2):e1002232.  Crino M, Herrera AMM, Ananthapavan J, et al. Modelled Cost-Effectiveness of a Package Size Cap and a Kilojoule Reduction Intervention to Reduce Energy Intake from Sugar-Sweetened Beverages in Australia. *Nutrients* 2017;9(9) doi: 10.3390/nu9090983 [published Online First: 2017/09/08]  Doran CM, Byrnes JM, Cobiac LJ, et al. Estimated impacts of alternative Australian alcohol taxation structures on consumption, public health and government revenues. *Medical Journal of Australia* 2013;199(9):619-22.  Lal A, Mantilla-Herrera AM, Veerman L, et al. Modelled health benefits of a sugar-sweetened beverage tax across different socioeconomic groups in Australia: A cost-effectiveness and equity analysis. *PLoS medicine* 2017;14(6):e1002326. doi: 10.1371/journal.pmed.1002326 [published Online First: 2017/06/28]  Lal A, Mihalopoulos C, Wallace A, et al. The cost-effectiveness of call-back counselling for smoking cessation. *Tobacco control* 2014;23(5):437-42. doi: 10.1136/tobaccocontrol-2012-050907 [published Online First: 2013/06/12]  Le LK, Barendregt JJ, Hay P, et al. The modelled cost-effectiveness of cognitive dissonance for the prevention of anorexia nervosa and bulimia nervosa in adolescent girls in Australia. *The International journal of eating disorders* 2017;50(7):834-41. doi: 10.1002/eat.22703 [published Online First: 2017/03/23]  Lee YY, Barendregt JJ, Stockings EA, et al. The population cost-effectiveness of delivering universal and indicated school-based interventions to prevent the onset of major depression among youth in Australia. *Epidemiology and psychiatric sciences* 2017;26(5):545-64. doi: 10.1017/s2045796016000469 [published Online First: 2016/08/12]  Lee YY, Veerman JL, Barendregt JJ. The cost-effectiveness of laparoscopic adjustable gastric banding in the morbidly obese adult population of Australia. *PloS one* 2013;8(5):e64965. doi: 10.1371/journal.pone.0064965 [published Online First: 2013/05/30]  Mantilla Herrera AM, Crino M, Erskine HE, et al. Cost-Effectiveness of Product Reformulation in Response to the Health Star Rating Food Labelling System in Australia. *Nutrients* 2018;10(5) doi: 10.3390/nu10050614 [published Online First: 2018/05/15]  Magnus A, Cadilhac D, Sheppard L, et al. The economic gains of achieving reduced alcohol consumption targets for Australia. *Am J Public Health* 2012;102(7):1313-9. doi: 10.2105/AJPH.2011.300453 [published Online First: 2012/05/19]  Magnus A, Moodie ML, Ferguson M, et al. The economic feasibility of price discounts to improve diet in Australian Aboriginal remote communities. *Australian and New Zealand journal of public health* 2016;40 Suppl 1:S36-41. doi: 10.1111/1753-6405.12391 [published Online First: 2015/07/01]  Moodie M, Haby MM, Swinburn B, et al. Assessing cost-effectiveness in obesity: active transport program for primary school children--TravelSMART Schools Curriculum program. *Journal of physical activity & health* 2011;8(4):503-15. [published Online First: 2011/05/21]  Moodie ML, Carter RC, Swinburn BA, et al. The cost-effectiveness of Australia's Active After-School Communities program. *Obesity (Silver Spring, Md)* 2010;18(8):1585-92. doi: 10.1038/oby.2009.401 [published Online First: 2009/11/07]  Moodie ML, Herbert JK, de Silva-Sanigorski AM, et al. The cost-effectiveness of a successful community-based obesity prevention program: the be active eat well program. *Obesity (Silver Spring, Md)* 2013;21(10):2072-80. doi: 10.1002/oby.20472 [published Online First: 2013/04/05]  Sampaio F, Barendregt JJ, Feldman I, et al. Population cost-effectiveness of the Triple P parenting programme for the treatment of conduct disorder: an economic modelling study. *European child & adolescent psychiatry* 2018;27(7):933-44. doi: 10.1007/s00787-017-1100-1 [published Online First: 2017/12/31]  Shih ST, Carter R, Heward S, et al. Economic evaluation of future skin cancer prevention in Australia. *Preventive medicine* 2017;99:7-12. doi: 10.1016/j.ypmed.2017.01.013 [published Online First: 2017/01/31]  Veerman JL, Zapata-Diomedi B, Gunn L, et al. Cost-effectiveness of investing in sidewalks as a means of increasing physical activity: a RESIDE modelling study. *BMJ open* 2016;6(9):e011617. doi: 10.1136/bmjopen-2016-011617 [published Online First: 2016/09/22] |
| *BODE^3^ evaluation published or in press in a peer reviewed journal 2010 to 2018 inclusive* |
| Blakely T, Kvizhinadze G, Karvonen T, Pearson AL, Smith M, Wilson N. Cost-effectiveness and equity impacts of three HPV vaccination programmes for school-aged girls in New Zealand. Vaccine 2014;**32**:2645–56.  Pearson A, van der Deen F, Wilson N, Cobiac L, Blakely T. Theoretical impacts of a range of major tobacco retail outlet reduction interventions: Modelling results in a country with a smokefree nation goal. Tobacco Control 2014;**24**(e1).  Pearson AL, Kvizhinadze G, Wilson N, Smith M, Canfell K, Blakely T. Is expanding HPV vaccination programs to include school-aged boys likely to be value-for-money: a cost-utility analysis in a country with an existing school-girl program. BMC Infect Dis 2014;**14**:351.  Blakely T, Cobiac LJ, Cleghorn CL, Pearson AL, van der Deen FS, Kvizhinadze G, Nghiem N, McLeod M, Wilson N. Health, Health Inequality, and Cost Impacts of Annual Increases in Tobacco Tax: Multistate Life Table Modeling in New Zealand. PLoS Med 2015;**12**(7):e1001856.  Blakely T, Collinson L, Kvizhinadze G, Foster R, Dennett E, Sarfati D. Cancer care coordinators in stage III colon cancer: a cost-utility analysis BMC Health Services Research 2015;**15**:306.  Nghiem N, Blakely T, Cobiac LJ, Pearson AL, Wilson N. Health and Economic Impacts of Eight Different Dietary Salt Reduction Interventions. PLoS One 2015;**10**(4):e0123915.  Webber-Foster R, Kvizhinadze G, Rivalland G, Blakely T. Cost-effectiveness analysis of docetaxel versus weekly paclitaxel in adjuvant treatment of regional breast cancer in New Zealand. Pharmacoeconomics 2015;**32**:707-24.  Collinson L, Kvizhinadze G, Nair N, McLeod M, Blakely T. Economic evaluation of single-fraction versus multiple-fraction palliative radiotherapy for painful bone metastases in breast, lung and prostate cancer. Journal of Medical Imaging and Radiation Oncology 2016;**60**(5):650-60.  Leung W, Kvizhinadze G, Nair N, Blakely T. Adjuvant Trastuzumab in HER2-Positive Early Breast Cancer by Age and Hormone Receptor Status: A Cost-Utility Analysis. PLoS Med 2016;**13**(8):e1002067.  Nair N, Kvizhinadze G, Blakely T. Cancer Care Coordinators to Improve Tamoxifen Persistence in Breast Cancer: How Heterogeneity in Baseline Prognosis Impacts on Cost-Effectiveness. Value Health 2016;**19**(8):936-44.  Nghiem N, Blakely T, Cobiac LJ, Cleghorn CL, Wilson N. The health gains and cost savings of dietary salt reduction interventions, with equity and age distributional aspects. BMC Public Health 2016;**16**(1):423.  Wilson N, Nghiem N, Eyles H, Mhurchu CN, Shields E, Cobiac LJ, Cleghorn CL, Blakely T. Modeling health gains and cost savings for ten dietary salt reduction targets. Nutrition Journal 2016;**15**:44.  Cleghorn CL, Blakely T, Kvizhinadze G, van der Deen FS, Nghiem N, Cobiac LJ, Wilson N. Impact of increasing tobacco taxes on working-age adults: short-term health gain, health equity and cost savings. Tob Control 2017.  McLeod M, Kvizhinadze G, Boyd M, Barendregt J, Sarfati D, Wilson N, Blakely T. Colorectal cancer screening: How health gains and cost-effectiveness vary by ethnic group, the impact on health inequalities, and the optimal age-range to screen. Cancer Epidemiology Biomarkers & Prevention 2017;**26**(9):1391-400.  Nghiem N, Cleghorn CL, Leung W, Nair N, Deen FSvd, Blakely T, Wilson N. A national quitline service and its promotion in the mass media: modelling the health gain, health equity and cost–utility. Tobacco Control 2017;**27**(4):434-41.  Pearson AL, Cleghorn CL, van der Deen FS, Cobiac LJ, Kvizhinadze G, Nghiem N, Blakely T, Wilson N. Tobacco retail outlet restrictions: health and cost impacts from multistate life-table modelling in a national population. Tob Control 2017;**26**:579–85.  Teng AM, Kvizhinadze G, Nair N, McLeod M, Wilson N, Blakely T. A screening program to test and treat for Helicobacter pylori infection: Cost-utility analysis by age, sex and ethnicity. BMC Infect Dis 2017;**17**(1):156.  van der Deen FS, Wilson N, Cleghorn C, Kvizhinadze G, Cobiac L, Nghiem N, Blakely T. Impact of five tobacco endgame strategies on future smoking prevalence, population health and health system costs: two modelling studies to inform the tobacco endgame. Tob Control 2017;**27**(3):http://dx.doi.org/10.1136/tobaccocontrol-2016-053585.  Wilson N, Kvizhinadze G, Pega F, Nair N, Blakely T. Home modification to reduce falls at a health district level: Modeling health gain, health inequalities and health costs. PLoS One 2017;**12**(9):e0184538.  Deverall E, Kvizhinadze G, Pega F, Blakely T, Wilson N. Exercise programmes to prevent falls among older adults: modelling health gain, cost-utility and equity impacts. Injury Prevention 2018;**10.1136/injuryprev-2016-042309**.  Jaine R, Kvizhinadze G, Nair N, Blakely T. Cost-effectiveness of a low-dose computed tomography screening programme for lung cancer in New Zealand. Lung Cancer 2020;**144**:99-106.  Petrović-van der Deen FS, Blakely T, Kvizhinadze G, Cleghorn CL, Cobiac LJ, Wilson N. Restricting tobacco sales to only pharmacies combined with cessation advice: a modelling study of the future smoking prevalence, health and cost impacts. Tobacco Control 2019;**28:**643-650.*  Cleghorn C, Wilson N, Nair N, Kvizhinadze G, Nghiem N, McLeod M, Blakely T. Health Benefits and Cost-Effectiveness From Promoting Smartphone Apps for Weight Loss: Multistate Life Table Modeling. JMIR Mhealth Uhealth 2019;**7**(1):e11118.*  Nghiem N, Leung W, Cleghorn C, Blakely T, Wilson N. Mass media promotion of a smartphone smoking cessation app: modelled health and cost-saving impacts. BMC Public Health 2019;**19**(1):283.*  Petrović-van der Deen F, Wilson N, Crothers A, Cleghorn C, Gartner CE, Blakely T. The potential health and cost impacts of legalizing domestic sale of vaporized nicotine products at a country level: A modelling study. Epidemiology 2019;**30**(3):396-404.* |

# Supplementary figures

Supplementary Figure 1: Net health system costs per 1000 total population for selected Australian and New Zealand health sector interventions


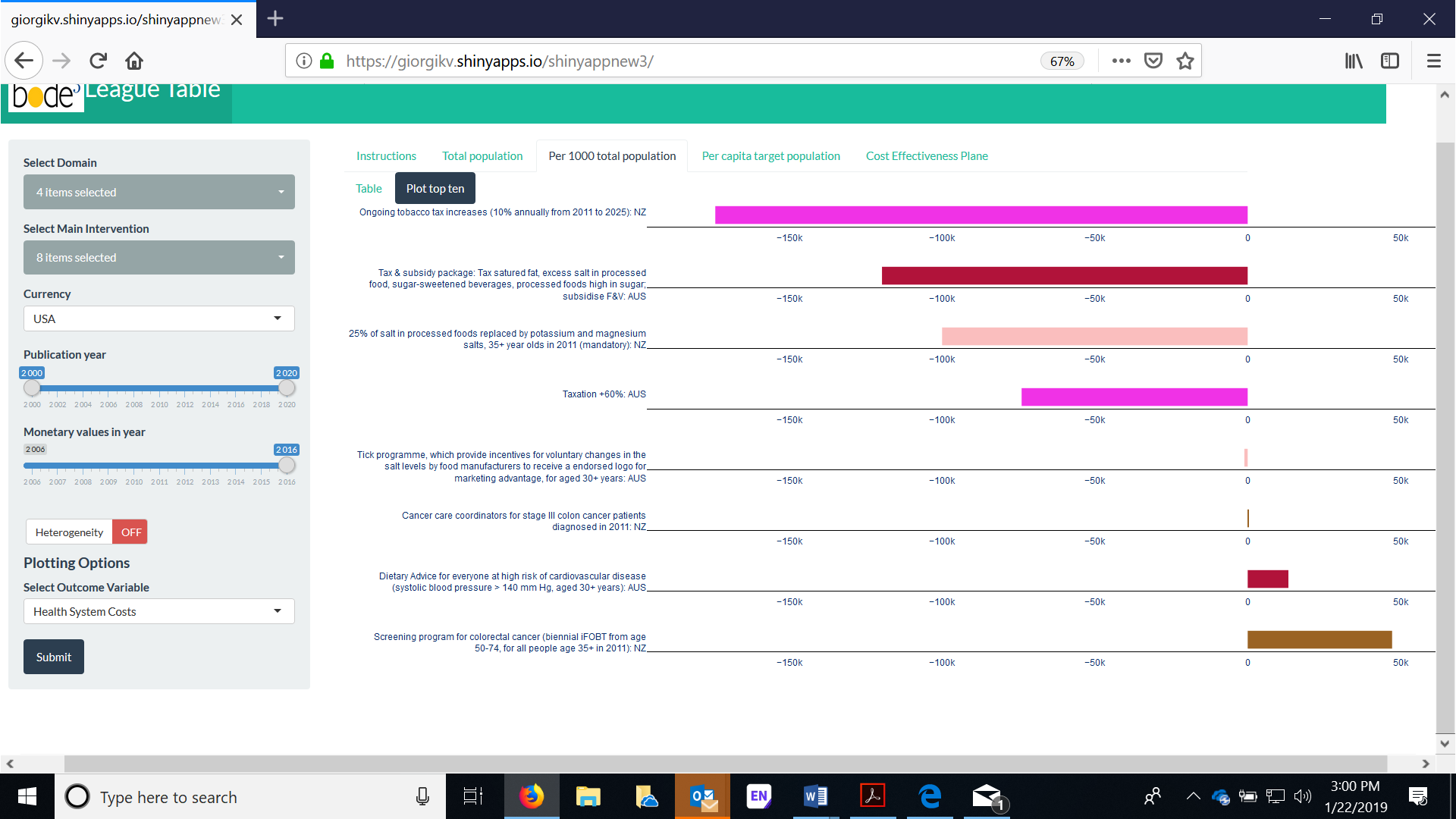


Supplementary Figure 2: HALYs gained per capita target population, for selected Australian and New Zealand health sector interventions


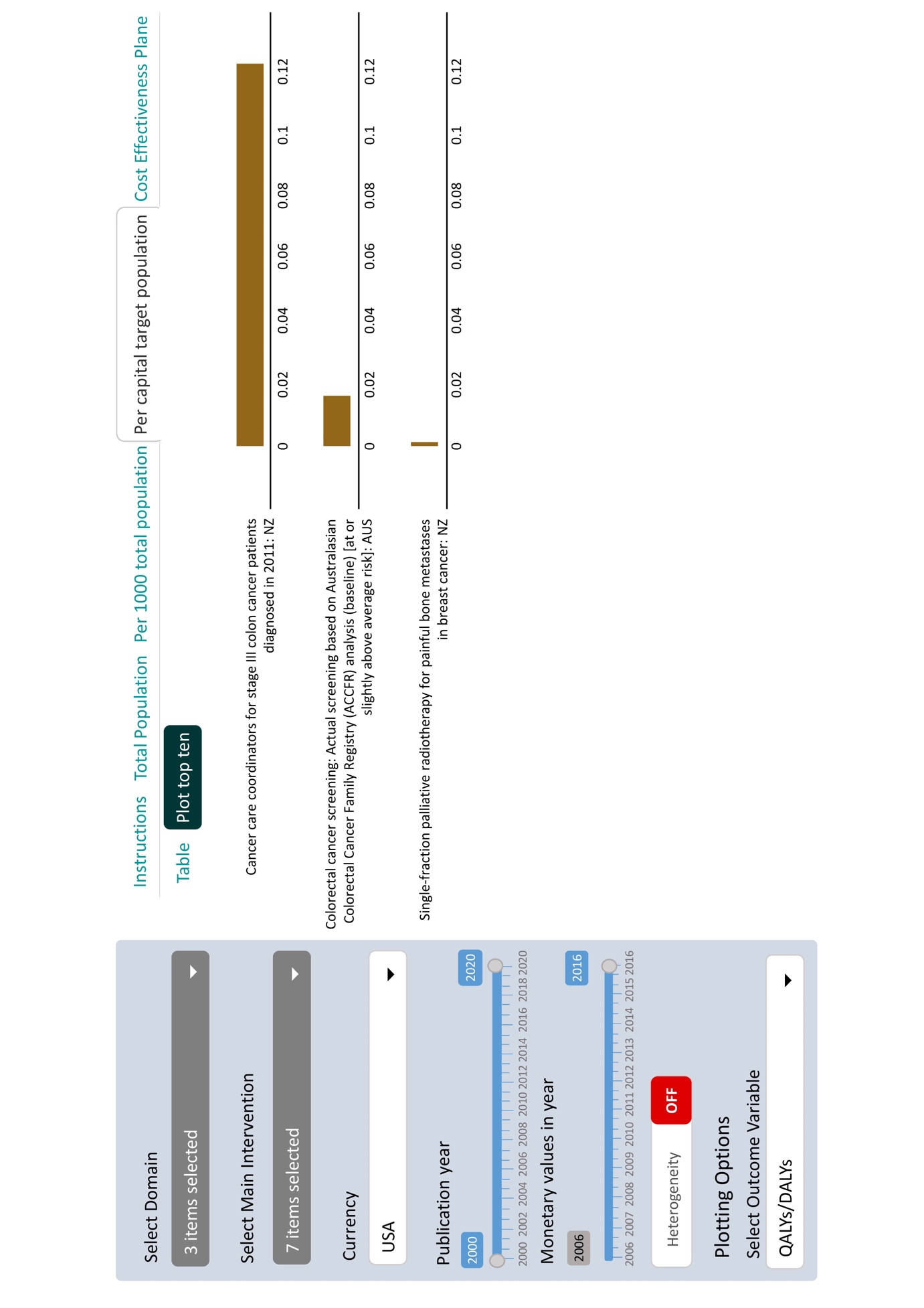


# Examples of (interactive) leagues tables that already exist

Several examples of leagues tables have been published in the last three decades. We conducted a literature search with the following search strategy (PubMed in 4 August 2017):

(league tables)

AND

((cost-effectiveness analysis) or (health economics) or (cost-benefit analysis) or (cost-utility analysis) OR (economic evaluation) or (economics))

This search resulted in 71 publications published between 1982 and 2017. A title and abstract review identified 41 studies original articles presenting league tables, methodological guidelines, theoretical and conceptual review, systematic reviews and critic letters. We also added 11 eligible articles found within the citations of these 41 studies. Un updated review with the same strategy on articles published between July 2017 and December 2020 (conducted in PubMed on 8 February 2021) found one additional paper. Those papers presenting actual examples of league tables, as opposed to guidelines or critiques, are shown in Table 3 below.

Supplementary Table 1: Examples of league tables published between 1985-2020

| **Authorship** | **Year** | **Region/ Country** | **Diseases/ Risk factor** | **Description** |
| --- | --- | --- | --- | --- |
| Williams  (1) | 1985 | United Kingdom | Cardiac diseases | Compares several cardiac and non-cardiac interventions by cost per QALY to discuss if the number of operations for coronary artery bypass grafting should be increased. |
| Chapman et al  (2) | 2000 | - | - | Compilation of 228 studies (1976-1997) and 647 intervention-comparator pairs, grouped by disease category and type of intervention. |
| George, Harrys & Mitchell (3) | 2001 | Australia | - | Compilation of 355 submissions to the Pharmaceutical Benefits Advisory Committee (PBAC) (1991-1996) to compare outcome measures: 26 CEA studies and 9 CUA (QALY gained). |
| Pinkerton et al (4) | 2001 | United States of America | Reduction of sexual transmission of HIV | Presents 15 studies, including multiples interventions (29 in total), using as outcome “infection averted”. |
| Hutubessy et al (5) | 2005 | Netherlands | Diabetes type 2 | *Stochastic* league table application using cost-effectiveness results of medical guidelines for patients in primary and secondary care. |
| Mortimer & Segal (6) | 2005 | Australia | Prevention or treatment of problem drinking and alcohol dependence | A league table with 16 interventions (competing and complementary), using QALY as an outcome, detailed comparator and target population. |
| Greenberg et al (7) | 2010 | 50% of the studies from USA | Cancer (Breast, colorectal and Haematologic) | Extraction of 242 studies (up to 2007) from Tufts Registry, summarises results by disease (presenting intervention description, comparator, target population, ICER and study rating). |
| Baeten et al (8) | 2010 | European  region (data from the Global Burden of Disease Studies) | Breast cancer | *Composite* league tables using the “usual approach”, target intervention, equity weighting and multicriteria decision analysis; by cancer stage and age group according to the standardized WHO CHOICE method. |
| Menzin et al (9) | 2011 | - | Chronic Kidney disease (CKD) | Systematic Review of 84 studies published between 1998-2008 (72 cost-utilities and 20 cost-effectiveness ratios) including studies from Tufts Registry and UK NHS Economic Evaluations database. |
| Dobbs et al (10) | 2014 | - | Obesity | Presents a list of interventions ranked by average cost per DALY (74 interventions grouped in 18 areas). |
| Nichols et al (11) | 2014 | Zambia (rural area) | HIV/AIDS | Uses *Stochastic* league table approach to compare epidemiological and CE impact of the use of pre-exposure prophylaxis (PrEP) in different scenarios of treatment initiation (per CD4 cell levels) to predict the optimal intervention per budget level. |
| Leigh & Granby (12) | 2016 | United Kingdom | Cancer | Uses data from NICE to create a league table with 74 cancer treatment interventions (several cancer forms), to estimate the Cancer Drug Fund (CDF) willingness to pay for cancer drugs. |
| Chiu et al (13) | 2017 | South Africa | HIV/AIDS | Comparison between a Conventional league table (modified to ICERs calculated between interventions in the table) with a league table with optimisation routine including the interaction between interventions, different levels of coverage of various interventions in HIV/AIDS. |
| Wilson et al (14) | 2019 | New Zealand | - | Compilation of 21 studies with cost-effectiveness analyses for New Zealand over the period 1 January 2010 to 8 October 2017. Study outcomes had to include cost-per QALY or life year (LY) gained, or cost per DALY averted. |

Apart from specific examples published Table 3, more comprehensive registries of CEA studies have been created and used with the diverse aims – including as league tables.

**Tufts Medical Center Cost-Effectiveness Analysis Registry (Tufts CEA Registry):** this registry started in 1976 and is a database of over 9,000 CUA covering a large variety of diseases and treatments that have been used as a source for many publications (e.g. two are cited in Table 3). The registry entries summarize and review published and original CUA articles (in English-language), using a standard auditing form to extract information on methodology, ICERs and utility weights. The review is performed by two trained readers: after an independent read and audit, the discrepancies are solved by consensus, and the disease classification is assigned by a clinician.

**Tufts Medical Center Global Health Cost-Effectiveness Analysis Registry (GH CEA Registry):** this registry, also hosted by Tufts Medical Center, is a repository of all peer-reviewed cost-per-DALY studies published since the 1990s. It includes 779 studies covering 2,548 unique interventions across 218 countries, covering a large variety of diseases and treatments.

**Australian Assessing Cost Effectiveness in Prevention Study (ACE-Prevention):** this project evaluated cost-effectiveness of 123 illness prevention measures and 27 treatment or infectious disease interventions. The studied risk factors included alcohol, tobacco, illicit drugs, physical activity, nutrition, blood pressure and cholesterol, bone mineral density; and diseases as cervical cancer, pre-diabetes, CKD, vision loss, osteoarthritis, influenza, HIV/AIDS, shingles and several mental health diseases. They were modelled for the Australian population using a standard protocol. A separate chapter was dedicated to the Australian Indigenous population. Results were expressed in cost per DALY averted and published in 2010.(15)

**United Kingdom Economic Evaluation Database (EED):** similar to the Tufts CEA registry, but collated by the University of York. Includes about 8000 evaluations.

**New Zealand Burden of Disease Epidemiology, Equity & Cost-Effectiveness Programme (BODE^3^) – Interactive League table:** this program was developed to estimate health and societal gains, costs, cost-effectiveness and equity impacts of health sector interventions (<http://www.otago.ac.nz/bode3>). The interventions can be compared and ranked in an interactive league table, by QALYs, costs and ICERS. Currently, there are available interventions for cancer, injury prevention, salt reduction and tobacco control. The results can be displayed with uncertainty and by different levels of heterogeneity (e.g. age, sex and ethnicity), and selected outcomes can be presented graphically.

# References

1. Williams A. Economics of coronary artery bypass grafting. Br Med J (Clin Res Ed). 1985;261(6491):326-9.

2. Chapman RH, Stone PW, Sandberg EA, Bell C, Neumann PJ. A comprehensive league table of cost-utility ratios and a sub-table of "panel-worthy" studies. Medical decision making : an international journal of the Society for Medical Decision Making. 2000;20(4):451-67.

3. George B, Harris A, Mitchell A. Cost-effectiveness analysis and the consistency of decision making: evidence from pharmaceutical reimbursement in australia (1991 to 1996). PharmacoEconomics. 2001;19(11):1103-9.

4. Pinkerton SD, Johnson-Masotti AP, Holtgrave DR, Farnham PG. Using cost-effectiveness league tables to compare interventions to prevent sexual transmission of HIV. AIDS (London, England). 2001;15(7):917-28.

5. Hutubessy RC, Niessen LW, Dijkstra RF, Casparie TF, Rutten FF. Stochastic league tables: an application to diabetes interventions in the Netherlands. Health economics. 2005;14(5):445-55.

6. Mortimer D, Segal L. Economic evaluation of interventions for problem drinking and alcohol dependence: cost per QALY estimates. Alcohol and alcoholism (Oxford, Oxfordshire). 2005;40(6):549-55.

7. Greenberg D, Earle C, Fang CH, Eldar-Lissai A, Neumann PJ. When is cancer care cost-effective? A systematic overview of cost-utility analyses in oncology. Journal of the National Cancer Institute. 2010;102(2):82-8.

8. Baeten SA, Baltussen RM, Uyl-de Groot CA, Bridges J, Niessen LW. Incorporating equity-efficiency interactions in cost-effectiveness analysis-three approaches applied to breast cancer control. Value in health : the journal of the International Society for Pharmacoeconomics and Outcomes Research. 2010;13(5):573-9.

9. Menzin J, Lines LM, Weiner DE, Neumann PJ, Nichols C, Rodriguez L, et al. A review of the costs and cost effectiveness of interventions in chronic kidney disease: implications for policy. PharmacoEconomics. 2011;29(10):839-61.

10. Dobbs R, Sawers C, Thompson F, Manyika J, Woetzel J, Child P, et al. Overcoming obesity: An initial economic analysis. 2014.

11. Nichols BE, Baltussen R, van Dijk JH, Thuma PE, Nouwen JL, Boucher CA, et al. Cost-effectiveness of PrEP in HIV/AIDS control in Zambia: a stochastic league approach. Journal of acquired immune deficiency syndromes (1999). 2014;66(2):221-8.

12. Leigh S, Granby P. A Tale of Two Thresholds: A Framework for Prioritization within the Cancer Drugs Fund. Value in health : the journal of the International Society for Pharmacoeconomics and Outcomes Research. 2016;19(5):567-76.

13. Chiu C, Johnson LF, Jamieson L, Larson BA, Meyer-Rath G. Designing an optimal HIV programme for South Africa: Does the optimal package change when diminishing returns are considered? BMC Public Health. 2017;17(1):143.

14. Wilson N, Davies A, Brewer N, Nghiem N, Cobiac L, Blakely T. Can cost-effectiveness results be combined into a coherent league table? Case study from one high-income country. Popul Health Metr. 2019;17(1):10.

15. Vos T, Carter R, Barendregt J, Mihalopoulis C, Veerman L, Magnus A, et al. Assessing Cost-Effectiveness in the Prevention (Ace-Prevention): Final Report. University of Queensland and Deakin University; 2010 September 2010.
